# Biofilm Formation in *Campylobacter concisus*: The Role of the *luxS* Gene

**DOI:** 10.3390/microorganisms12010046

**Published:** 2023-12-27

**Authors:** Mohsina Huq, Syeda Umme Habiba Wahid, Taghrid Istivan

**Affiliations:** 1 School of Science, STEM College, RMIT University, Bundoora, Melbourne, VIC 3083, Australia; 2 Department of Medical Laboratories, College of Applied Medical Sciences, Qassim University, Buraydah 51452, Saudi Arabia

**Keywords:** *Campylobacter concisus*, *luxS*, bacterial biofilms

## Abstract

*Campylobacter concisus* is a bacterium that inhabits human oral cavities and is an emerging intestinal tract pathogen known to be a biofilm producer and one of the bacterial species found in dental plaque. In this study, biofilms of oral and intestinal *C. concisus* isolates were phenotypically characterized. The role of the *luxS* gene, which is linked to the regulation of biofilm formation in other pathogens, was assessed in relation to the pathogenic potential of this bacterium. Biofilm formation capacity was assessed using phenotypic assays. Oral strains were shown to be the highest producers. A *luxS* mutant was created by inserting a kanamycin cassette within the *luxS* gene of the highest biofilm-forming isolate. The loss of the polar flagellum was observed with scanning and transmission electron microscopy (SEM and TEM). Furthermore, the *luxS* mutant exhibited a significant reduction (*p* < 0.05) in biofilm formation, motility, and its expression of *flaB*, in addition to the capability to invade intestinal epithelial cells, compared to the parental strain. The study concluded that *C. concisus* oral isolates are significantly higher biofilm producers than the intestinal isolates and that LuxS plays a role in biofilm formation, invasion, and motility in this bacterium.

## 1. Introduction

*Campylobacter concisus* is a fastidious, hydrogen-requiring bacterium commonly found in the human oral cavity and is an emerging gastroenteritis pathogen. It has been associated with acute intestinal diseases [1,2] and chronic intestinal diseases, such as Crohn’s disease (CD) in children [3] and ulcerative colitis (UC) in adults [4]. It has been isolated from both healthy and diseased periodontal sites, although healthy sites have been shown to harbour higher bacterial loads [5]. It is also associated with gingivitis [6] and periodontitis [7] and was identified in saliva from inflammatory bowel disease (IBD) patients [8]. Despite its fastidious nature and sensitivity to environmental oxygen, *C. concisus* can survive in the human oral cavity, and its survival strategy is likely to be associated with the formation of biofilms at this site. *C. concisus* has been shown to be capable of producing biofilms on a range of different surfaces, including glass, stainless steel, and polystyrene [9,10]. More recently, oral strains were shown to have a significantly greater capacity for biofilm formation compared to faecal isolates (*p* < 0.03), with a strong negative correlation between motility and biofilm formation [11].

Biofilms are made from complex aggregations of planktonic microorganisms that serve to protect the resident individuals from hostile environments and are covered with a protective layer consisting of a mixture of extracellular polymeric substances (EPS) secreted by the cells established within the biofilm [12,13]. Biofilm formation requires a concerted mechanism regulated by numerous environmental signals [14] and has been linked to the LuxS enzyme in oral pathogens such as *Streptococcus mutans*, *Streptococcus intermedius*, and *Eikenella corrodens* [15,16,17]. Several studies of oral bacteria have provided evidence that LuxS is involved in interspecies signal responses among oral bacteria and, consequently, may play important roles in the development of virulence and dental biofilms [18,19]. The LuxS enzyme synthesizes autoinducer 2 (AI-2) as a by-product, which is required for quorum-sensing (QS). QS is a population-dependent signalling mechanism that involves both inter- and intra-species production and detection of extracellular signalling molecules [20]. In many Gram-negative bacteria, QS is based upon the signalling molecule homoserine lactone (HSL), which is classified as an autoinducer-1 (AI-1). Furthermore, an alternative quorum-sensing system in Gram-negative and Gram-positive bacteria is mediated by furanosyl borate diester, referred to as AI-2. LuxS is responsible for the synthesis of the AI-2 precursor, 4,5-dihydroxy-2,3-pentanedione (DPD) [20]. Therefore, LuxS has been of interest to researchers because it plays an important role in the development and spatial organization of oral biofilms [19,21,22]. The presence of *luxS* in *Campylobacter* spp. has been investigated by several research teams [23,24]. The *luxS* gene, or its homologue, was detected in *C. jejuni*, *C. coli*, *C. upsaliensis*, and *C. fetus* genomes with PCR or Southern blotting hybridization [25]. Reeser et al. [23] reported that *flaAB* and *luxS* mutants of *C. jejuni* produced a significantly reduced amount of biofilm in comparison to that of wild-type strains. The role of LuxS in the motility of *C. jejuni* was demonstrated in several studies [26,27,28], and the downregulation of major flagellin genes (*flaA* and *flaB*) was observed [29].

To our knowledge, no study has been performed to investigate *luxS* in *C. concisus* and to assess its association with biofilm formation, motility, and other virulence properties. In this study, we investigated the phenotypic characteristics of *C. concisus* biofilms and the role of *luxS* in biofilm formation and bacterial interaction with host cells.

## 2. Materials and Methods

### 2.1. Bacterial Strains and Plasmids

A total of 34 *C. concisus* isolates were included in the study. A total of 15 intestinal isolates (RCH 3–RCH 8, RCH 11, RCH 12, RCH 15, RCH 19, RCH 20, RCH 23–RCH 26) were from children suffering from mild to severe bloody diarrhoea and isolated using the Cape Town Protocol [30] at the Royal Children’s Hospital, Melbourne, Australia between June 1993 and June 1995; 19 oral isolates (RMIT-O2–RMIT-O15, RMIT-O17, RMIT-O22, RMIT-O23, RMIT-O27, and RMIT-O33) were from gum swabs of volunteers from RMIT University, Melbourne, Australia between February and December 2014 The isolates used in this study (intestinal and oral) were classified into two molecular groups, genomospecies A and genomospecies B, using PCR amplification and sequencing of the 23S rRNA gene, following the method of Istivan et al. [31]. The reference strains *C. concisus* ATCC 51561 and *C. concisus* ATCC 51562, *C. jejuni* NCTC 11168, *C. jejuni* 81116 (NCTC 11828), and a non-*Campylobacter* strain, *Escherichia coli* ATCC 25922, were included as control strains. *C. concisus* RMIT-O17 oral isolate, the highest biofilm producer, was selected to create a *luxS* null mutant by inserting a kanamycin resistance gene. *E. coli* DH5α was used for cloning purposes.

### 2.2. Bacterial Growth Conditions

All *Campylobacter* spp. strains were grown on Columbia blood agar base (Oxoid, Thermo Fisher Scientific Inc. Hampshire, UK) supplemented with 5–7% (*v*/*v*) defibrinated horse blood (HBA), incubated at 37 °C for 2–4 days in anaerobic jars flushed with a gas mixture consisting of 6% O_2_, 8% CO_2_, 6% H_2_, and 80% N_2_. Liquid cultures of all *Campylobacter* spp. strains were grown in Brucella broth (Oxoid, Thermo Fisher Scientific Inc. Hampshire, UK), supplemented with 2% yeast extract, and incubated in jars with the same gas mixture at 37 °C for 2–4 days. *E. coli* strains were grown on nutrient agar (NA) for 18 h at 37 °C or in nutrient broth and grown for 18 h at 37 °C on an orbital shaker at 220 rpm. *E. coli* clones were cultured on Luria–Bertani agar (LBA) or in Luria–Bertani (LB) broth with appropriate antibiotics, under the same conditions. The identity of the *C. concisus* strains was confirmed through biochemical reactions such as Gram reaction, oxidase production, and catalase production following standard methods [32].

### 2.3. Crystal Violet (CV) Assay to Assess Biofilm-Formation by C. concisus Isolates

*C. concisus* isolates were grown on HBA at 37 °C in hydrogen-enriched microaerophilic conditions. The biofilm assay was performed in 96-well microtitre plates. Each well was inoculated with 200 µL of diluted *C. concisus* cell suspension (~0.2 × 10^8^ CFU) in Brucella broth. Plates were incubated at 37 °C in the same gas mixture described above for 24 h, 48 h, 72 h, and 96 h. Following incubation, biofilm formation was determined by staining with a 0.1% crystal violet (CV) solution, according to the method described by Reeser et al. [23]. The absorbance at 570 nm (A_570_) was determined using a microplate reader (Polarstar Omega microplate reader, BMG Labtech, Ortenberg, Germany) to indirectly determine the amount of biofilm. *C. concisus* ATCC 51561 and ATCC 51562 and *C. jejuni* and *C. coli* were also included to compare the biofilm formation. The OD reading at 570 nm was interpreted as follows: <0.1: no biofilm, 0.1–0.5: low, 0.5–1.0: moderate, >1.0: high. Each experiment was performed three times independently and in technical triplicates. In each experiment, a negative control (broth without bacterial inoculum) was included to account for the non-specific binding of the CV stain to the 96-well plates. Statistical analysis was performed using the Mann–Whitney test: two-sample assuming unequal variances in GraphPad Prism 8.4.2 for Windows (GraphPad Software, La Jolla, CA, USA, www.graphpad.com).

### 2.4. Phenotypic Characterization of C. concisus Biofilm

One oral *C. concisus* RMIT-O17 isolate was selected for further investigation of the biofilm by microscopy. Each experiment was performed three times independently and in technical triplicates.

#### 2.4.1. Phase Contrast Microscopy

Cell suspension of biofilm-producing *C. concisus* was prepared and incubated in the same gas mixture described above. Plates were incubated for 24, 48, 72, 96, or 120 h to observe the growth and development of biofilms attached to the polystyrene surface phase contrast under 400× magnification using an Olympus CKX41 microscope.

#### 2.4.2. Confocal Laser Scanning Microscopy (CLSM)

Bacterial suspensions were prepared and dispended into 6-well flat bottom culture plates with a sterile coverslip (~0.5 cm × 0.5 cm) embedded within the well. After incubation, the biofilms were stained using a LIVE/DEAD BacLight bacterial viability kit (Invitrogen, Carlsbad, CA, USA) (3.35 μM of Syto-9 and 20 μM of Propidium iodide [PI]) according to the manufacturer’s instructions. The biofilms were examined at 400× magnification and scanned first at 482 nm and then at 635 nm with a Nikon Eclipse Ti inverted microscope, (Nikon Instruments Inc., Melville, NY, USA) equipped with Nikon A1R Fast Laser Scanning Confocal system.

#### 2.4.3. Scanning Electron Microscopy (SEM)

Bacterial suspensions were prepared and dispended into 6-well flat bottom culture plates containing round glass coverslips. Once the biofilms had grown, the coverslips were removed and fixed with Karnovsky fixative in 2% paraformaldehyde and 2.5% glutaraldehyde in 0.1 M sodium cacodylate buffer (pH 7.0) (Electron Microscopy Sciences, Hatfield, PA, USA) for 30 min at room temperature. After fixation, samples were washed with the same buffer 3 times and then dehydrated in a series of ethanol solutions (50%, 70%, 90%, 95%, and 2× 100%). They were subsequently air-dried in a ventilated covered container for at least 1 h and sputter-coated with a thin layer of gold in an Edwards S150 sputter coater. Next, the coverslip was mounted on SEM stubs of a scanning electron microscope (Quanta 200 scanning electron microscope, EM; FEI Co., Inc., Hillsboro, OR, USA) and used at high vacuum in the secondary electron imaging mode to observe the biofilms at 2500×, 10,000×, and 20,000× magnifications.

#### 2.4.4. Transmission Electron Microscopy (TEM)

To examine *C. concisus*, bacterial cells were stained with 1% phosphotungstic acid (pH 7.0). In the negative staining procedure, only the background of the grid covered with a carbon-coated parlodion film (Yuanjiou Environment Technology, Tainan, Taiwan) is stained but not the bacteria. In brief, a 20 μL sample drop (~10^8^ CFU) was loaded on a carbon-coated grid and allowed to soak for 2 min before gently washed with distilled water. Excess water was removed with filter paper. Then, the grid was negatively stained with one drop of 1% phosphotungstic acid (pH 7.0) (Sigma-Aldrich, St. Louis, MO, USA) and left for 1 min. Excess stain was removed by touching the edge of the grid with a piece of Whatman filter paper. After staining, the sample was rinsed twice with distilled water and dried before examining the grid under transmission electron microscopy (JEOL, Tokyo, Japan).

### 2.5. Genomic DNA Extraction

Bacterial genomic DNA was purified using the Wizard Genomic DNA Purification Kit (Promega, Alexandria, NSW, Australia). The concentration and purity of gDNA were estimated using a Biophotometer (Eppendorf, Macquarie Park, NSW, Australia). The extracted DNA was stored at −20 °C.

### 2.6. RNA Extraction

*C. concisus* cells were grown on Columbia blood agar plates supplemented with 5% defibrinated horse blood and 0.4% Na-fumarate. Cells were harvested, washed, and, finally, resuspended into 100 mL of nuclease-free water. RNA was extracted using the ISOLATE II RNA Mini Kit (Bioline, Meridian Bioscience, Lukenwalde, Germany) following the manufacturer’s instructions. Extracted RNA was treated using a Turbo DNA-free^TM^ kit (Life Technologies, Melbourne, Australia) to remove residual genomic DNA. The RNA concentration was estimated using a NanoDrop 2000 spectrophotometer (Thermo Fisher Scientific, Wilmington, DE, USA).

### 2.7. PCR of luxS in C. concisus

PCR was used to detect the presence of *luxS* in *C. concisus* isolates. A primer set was designed from the sequence of *C. concisus* available on the NCBI website. The PCR amplification of the *luxS* in *C. concisus* was performed using primer pair 1 (Table 1) to produce a 309 bp product. The reference strains *C. jejuni* NCTC 11168, *C. jejuni* 81116 (NCTC 11828), *C. coli* NCTC 11366, and *C. mucosalis* ATCC 43264 are known to carry the gene. A non-*Campylobacter* strain, *E. coli* ATCC 25922, was included as a negative control in the PCR. Primers were designed using Clone Manager software (Sci-Ed Software, version 7.11).

### 2.8. Construction of Insertional luxS Mutant and Complementary Plasmid

*C. concisus* RMIT-O17 was selected to create a *luxS* null mutant by inserting a kanamycin resistance gene. A set of degenerate primers (Table 1: primer pair 2, 2a and 2b) were designed from the flanking regions of *luxS* to amplify the whole *luxS* (516 bp), including the promoter region. Primer pairs were designed with *Kpn*I and *Sac*I restriction sites, respectively. The amplified product (1380 bp) was cloned into a pCR2.1-TOPO vector using the TOPO TA Cloning Kit (Invitrogen). The insert was confirmed by sequencing the plasmid pCR2.1-TOPO with the insert using M13 forward and reverse primers. After sequence confirmation, the insert was moved to the pBluescript SKII vector using the introduced *Kpn*I and *Sac*I sites. The ligated product was electro-transformed into electrocompetent *E. coli* DH5α. The newly constructed plasmid pBK*luxS* was confirmed through restriction enzyme analysis and PCR. There were no suitable restriction sites present within the sequence of the *luxS* of *C. concisus* O17 for insertion of a kanamycin resistance cassette; therefore, inverse PCR (Table 1: primer pair 3) was used to introduce a unique *BamH*I restriction site and to delete part of the *luxS* (136 bp). The kanamycin cassette (1.4 Kb) was extracted from plasmid pMW2 through restriction enzyme digestion with *Bam*HI [33]. The purified inverse PCR product was digested with *Bam*HI. Then, the products were ligated with a kanamycin cassette of the pMW2 vector. The newly constructed plasmid pBK*luxS*kan was electro-transformed into *E. coli* DH5α cells and grown with kanamycin (50 µg/mL) selection. Transformation of the pBK*luxS*kan into *C. concisus* RMIT-O17 was performed by natural transformation.

To complement the knocked-out gene, an 8 kb shuttle vector containing native cryptic *C. concisus* plasmid and *luxS,* along with an *E. coli* vector, was constructed to complement the function of LuxS in the mutant strain.

### 2.9. Natural Transformation of pBKluxSkan in C. concisus

The transformation was performed on an agar surface. For transformation on the agar surface, *C. concisus* RMIT-O17 was grown on Mueller–Hinton agar (MHA) for 24 h in microaerophilic conditions. Cells were collected in Mueller–Hinton broth (MHB) and then spread on a fresh MHA plate at about 5 × 10^8^ cells per plate and incubated for 6 h. After incubation, aliquots of plasmid DNA (10 µg in TE buffer) were spotted directly onto the MHA surface without additional mixing or spreading, and incubation was continued for 5 h. The cells were harvested in 1 mL of MHB from an MHA plate or test tubes plated out on HBA plates containing kanamycin (50 µg/mL). The plate was incubated in microaerophilic conditions for 3 d at 37 °C. The colonies were confirmed using colony PCR.

### 2.10. Whole Genome Sequencing and Assembly

Sequencing of RMIT-O17 and Δ*luxS*-O17 genomes was performed using an Illumina MiSeq sequencer at RMIT University. The libraries were prepared using a Nextera XT DNA library preparation kit (Illumina, San Diego, CA, USA, 2012) following the manufacturer’s instructions. The MiSeq^®^ Reagent Kit v3 (2 × 300 bp paired-end reads) was used to perform the run on the Illumina MiSeq. The de novo assembly of RMIT-O17 and Δ*luxS*-O17 was performed using the A5-miseq pipeline [34]. The assembled files were submitted to the Rapid Annotation using Subsystem Technology (RAST) [35], a service for annotating bacterial and archaeal genomes in relation to gene definition and annotation for individual assemblies. The gene of interest, *luxS*, was confirmed with homology BLAST searches of the NCBI database.

### 2.11. Motility Assay

The motility of *C. concisus* strains was investigated at 37 °C on semi-solid agar plates using a modification of a method described by Adler et al. [36]. Either semi-solid BB containing 0.4% agar (BBA 0.4%) or MH containing 0.4% agar (MHA 0.4%) were used for this test. Overnight cultures of *C. concisus* strains were adjusted to 10^8^ CFU/mL, and 3.8 µL of this suspension was dropped on semi-solid BBA or MHA. After 72 h incubation at 37 °C in microaerophilic conditions, the diameters of the growth were measured. Growths of mutants were normalized to the wild-type diameter (100%). The results reported are the median of six independent assays.

### 2.12. Adhesion and Invasion Assay

Adherence and invasion assays of *C. concisus* strains were performed using INT407 cells, as previously described [37]. Briefly, cells were cultured, harvested, and counted. The cell line was seeded at a concentration of 2 × 10^5^ cells per well and incubated overnight. Before commencing the assay, media were aspirated from each well, and the cells were washed 3 times with PBS. To each well, 100 μL of ~10^8^ bacterial cells (MOI ~50–100 bacteria per tissue culture cell) along with 400 μL of DMEM/FBS was added, and the culture trays were incubated at 37 °C in 5% CO_2_. Each assay was performed in triplicate and repeated three times on different days.

For the adhesion assay, tissue culture trays were incubated for 6 h, as described above. Wells were then washed 3 times with PBS. Tissue culture cells were lysed by the addition of 100 μL of 0.25% Triton X-100 into each well and incubation at 37 °C for 15 min. After pipetting 800 μL of PBS into each well, 100 μL was subjected to a serial 10-fold dilution before being plated out on HBA plates. Plates were incubated for 48–72 h at 37 °C in the presence of H_2_ and the bacteria enumerated.

For the invasion assay, tissue culture trays were incubated for 6 h. The contents of each well were washed 3 times with PBS before 500 μL of 400 μg/mL of gentamicin solution was added. Culture trays were incubated at 37 °C in 5% CO_2_ for 1 h to kill any extracellular bacteria. Wells were again washed thrice with PBS, and intracellular bacteria were released with Triton X-100, as mentioned above. Dilutions were plated and counted as outlined above. The invasive index was calculated using the formula described by Larson et al. [38].

### 2.13. Expression of Flagellin Gene flaB by RT-qPCR

Complementary DNA was generated using the RNA template in the Tetro cDNA Synthesis Kit (Bioline, Meridian Bioscience, Lukenwalde, Germany). The expression of the flagellin gene (*flaB*) was performed using the following primers: flaB-F (5′-CAAACAGCTGCAGATGAC-3′) and flaB-F (5′-GTCAAGCTCCTCCATTAGAC-3′). The ATP synthase F1 α-subunit gene (*atpA*) was used as a house-keeping gene for qPCR (Primer atpA-F* 5′-TGGCGCTATGGACTACACAA-3′ and atpA-R* 5′-TCAAAGATCCAGCGCCTAGT-3′). All PCR reactions were performed in a Biorad CFX Connect real-time PCR detection system (Bio-Rad Laboratories, Hercules, CA, USA). *C. concisus* ATCC 51562 was included as a control in this experiment. GraphPad Prism 8.4.2 was used for statistical analysis to calculate the mean and standard deviation and to generate the graphs. A change of more than 2 folds relative to control was considered a real change in expression, and further statistical analysis using an unpaired *t*-test was performed; a value of *p* < 0.05 was considered statistically significant.

## 3. Results

### 3.1. Screening of Biofilm-Forming C. concisus through Quantitative Crystal Violet (CV) Assay

Biofilm assays were carried out under various conditions, such as different media, inoculum, and time, to determine the optimum experimental conditions for *C. concisus*. The highest amount of biofilm was obtained using an inoculum of ~10^8^ CFU/mL in Brucella (BB) broth for 4 days.

**Figure 1:**
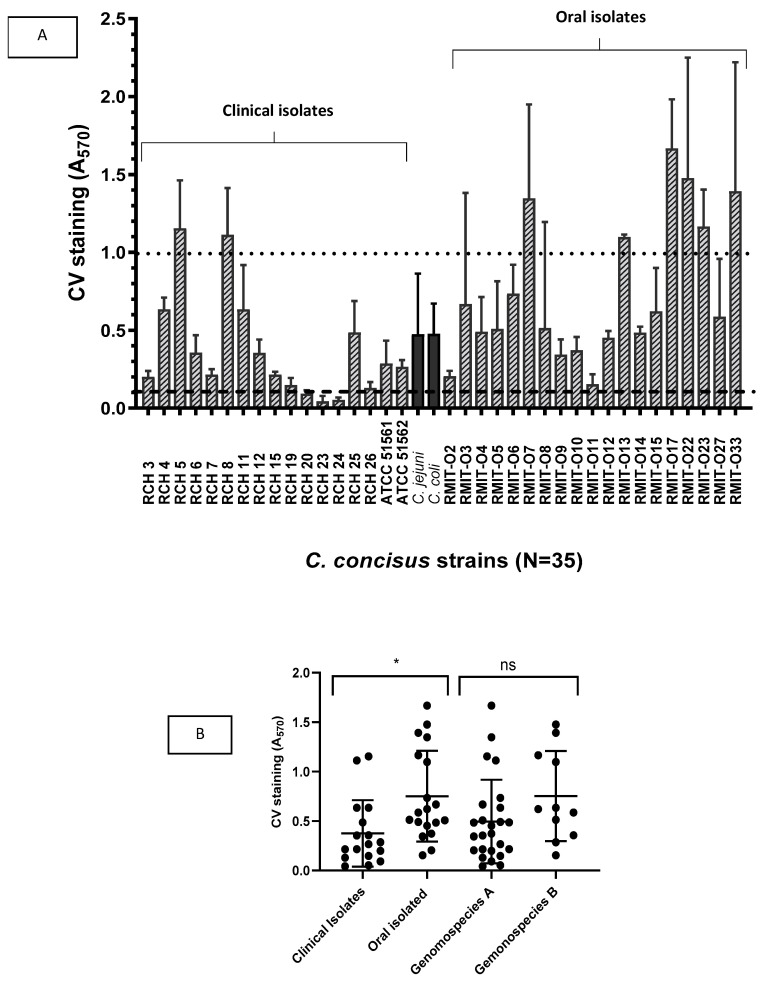
(**A**) Crystal violet quantitative assay for screening of biofilm formation by 35 *C. concisus* strains isolated from patients with gastritis and oral cavity of healthy persons. The black solid bars represent non-*C. concisus* control strains and the striped bars represent *C. concisus* isolates (oral and clinical). Biofilm-forming capacity varies significantly amongst isolates (Kruskal–Wallis; *p* < 0.05). *C. jejuni* and *C. coli* were used as control strains. The OD reading < 0.1 was the cut-off value for biofilm production, and OD value > 1.0 was considered as high at 570 nm. The results represent mean values and standard errors of three independent experiments. Each experiment was performed three times independently and in biological/technical triplicates. (**B**) Comparison between different groups of *C. concisus*. Oral isolates exhibit a significant amount of biofilm-forming potential than clinical isolates (*p*-value = 0.0354). However, there was no significant difference between the two genomospecies A and B (*p-*value = 0.1453). * *p* ≤ 0.05, ns = not significant.

Intestinal and oral *C. concisus* were screened for biofilm formation using the CV assay. Among 15 clinical isolates, 11 produced biofilms. RCH 20, 23, and 24 did not form any biofilm (Figure 1). RCH 3, RCH 6, RCH 7, RCH 12, RCH 15, RCH 19, RCH 25, and RCH 26 formed low amounts of biofilm. RCH 4 and RCH 11 formed moderate levels, and RCH 5 and RCH 8 formed a high amount of biofilm. *C. concisus* ATCC 51561, ATCC 51562, and *C. jejuni* and *C. coli* produced low amounts of biofilms. All oral isolates were able to form biofilms. Among the 19 oral *C. concisus*, RMIT-O2, RMIT-O4, RMIT-O9, RMIT-O10, RMIT-O11, RMIT-O12, and RMIT-O27 produced low amounts of biofilm. RMIT-O3, RMIT-O5, RMIT-O6, RMIT-O8, and RMIT-O15 produced moderate levels, and RMIT-O7, RMIT-O13, RMIT-O17, RMIT-O22, RMIT-O23, and RMIT-O33 produced high levels of biofilms. RMIT-O17 was the highest biofilm former. A comparison of strains isolated from oral and clinical sites revealed that oral isolates exhibited significantly greater biofilm-forming capacity compared with clinical isolates (Mann–Whitney; *p* = 0.0354).

### 3.2. Phenotypic Characterization of C. concisus Biofilm

Development of *C. concisus* biofilm was observed with phase contrast, CLSM and SEM continuously for 5 days. For this experiment, the highest biofilm producer strain, RMIT-O17, was selected.

**Figure 2:**
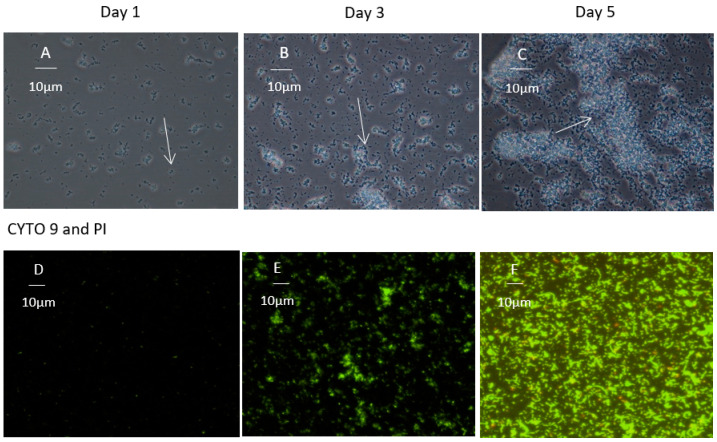
Biofilm development by *C. concisus* RMIT-O17 examined by phase contrast and CLS microscopy. Each panel represents every other day in biofilm development. (**A**) Day 1, bacterial attachment, the initial event in biofilm development, planktonic bacteria are attached to the surface (indicated with an arrow). (**B**) Day 3, cell clusters matured and embedded in the EPS matrix (indicated with an arrow). (**C**) Day 5, cell clusters are thickened and forming void spaces (indicated with an arrow). (**D**–**F**) Biofilms on glass cover slip observed by CLSM at different times of incubation stained with SYTO-9 and PI.

Phase contrast microscopy of *C. concisus* RMIT-O17 biofilm showed several morphological arrangements (Figure 2A–C). After day 1 of incubation, planktonic bacterial cells were found to be attached at the bottom of the plate; small cell clusters were visualized as multiple cells in contact with one another (Figure 2A). This is the first stage of biofilm formation. On day 3, it was observed that cell clusters became progressively larger (Figure 2B). Multi-layered biofilm was observed, indicating maturation. During biofilm maturation (or maturation stage), the majority of the cells are segregated within cell clusters. The cell clusters reach their maximum dimensions, and they become displaced from the edge of the well. At day 5 (Figure 2C), cell clusters were observed to undergo alterations in their structure due to the dispersion of bacterial cells from their interior. These bacterial cells were motile and were observed to swim away from the inner portions of the cell cluster through openings in the cluster and enter the bulk liquid. In addition, other cells remaining within the void space were motile. The ability of bacteria to swim freely within the void spaces, as observed through microscopy, indicated the absence of dense polymers or other gel-like material in the void space.

Different developmental arrangements of the biofilm of RMIT-O17 were observed on days 1, 3, and 5 by CLSM (Figure 2D–F). After day 1 of incubation, only a few cells with green colour emissions were observed following the SYTO-9 staining, indicating that only live single cells were attached to the coverslip (Figure 2D). There was no red-light emission from PI on day 1, indicating that there were no dead cells on the coverslip (Figure 2E). This finding of single live cells on the coverslip was in agreement with the findings on day 1 from phase contrast microscopy (Figure 2A). After 3 days incubation, there were some small aggregates of live cells stained with SYTO-9 (Figure 2E). However, there was still no red colour emission from PI after 3 days of incubation, indicating that only live cells were present in the biofilm. After 5 days of incubation, a larger aggregation of cells emitting both red and green colour indicated both live and dead cells (Figure 2F), showing that a mature biofilm of *C. concisus* is a mixture of live and dead bacterial cells.

### 3.3. Molecular Detection of luxS in C. concisus

The PCR amplification experiments were carried out with 15 intestinal isolates and 19 oral isolates of *C. concisus* using the primer set 4 (FCCluxS/RCCluxS in Table 1). The primer pair successfully amplified products of 309 bp with all *C. concisus* isolates.

### 3.4. Construction of C. concisus ΔluxS Mutant, Complementary Plasmid, and Whole Genome Sequence Analysis

*C. concisus* RMIT-O17 was selected for the generation of the *luxS* knock-out because it produced the greatest amount of biofilm. The kanamycin cassette was successfully inserted within *luxS*. It was confirmed through colony PCR using the FCCluxS/RCCluxS primer set, and the mutant Δ*luxS*-O17produced a 1.7 kb PCR product. The PCR product was sequenced, which confirmed that the kanamycin cassette was inserted, and the orientation was correct.

Unfortunately, with many complementation attempts, the 8 kb *luxS* complementary plasmid could not be inserted into Δ*luxS*-O17 successfully. Therefore whole genome sequencing of RMIT-O17 and of Δ*lux*S-O17 was performed to identify that the kanamycin cassette was inserted only within *luxS* and not anywhere else. *luxS* and the WGS of Δ*lux*S-O17 aligned by NCBI BlastN. In the result, only one copy of *luxS* was found in scaffold 2 of the WGS with the same flanking region as *luxS* of RMIT-O17. When the alignment was performed, we found 136 nucleotides were replaced by 1404 bp kanamycin cassette by homologous recombination.

### 3.5. Phenotypic Characterization of the ΔluxS Mutant

**Figure 3:**
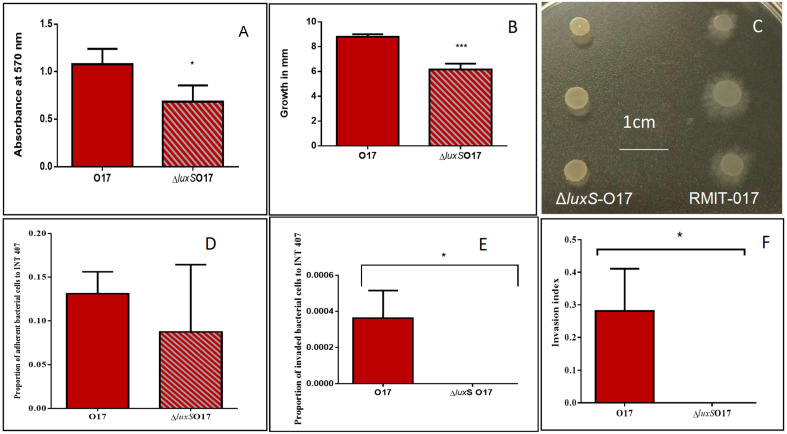
Comparisons between RMIT-O17 and Δ*lux*S-O17 by different virulence properties. The solid red bar represents RMIT-O17, and the striped red bar represents the Δ*luxS*-O17. All experiments were performed three times in triplicate, and error bars represent the standard error of the mean. (**A**) Significantly reduced biofilm formation by Δ*luxS*-O17. RMIT-O17 and Δ*lux*S-O17 were subjected to a standard biofilm CV assay (*p* < 0.02) (by *t*-test). (**B**) Reduced motility by Δ*lux*S-O17 by motility assay on semi-solid agar (0.4% BBA) shown in a graph. The growth diameter was measured after 72 h incubation on semi-solid agar. (**C**) Motility assay of RMIT-O17 and Δ*lux*S-O17 on semi-solid agar (0.4% BBA) showing reduced motility of the mutant. (**D**) Adherence of RMIT-O17 and Δ*lux*S-O17 to INT 407 epithelial cells. Adherence is expressed as the percentage of the inoculum that remained associated with the epithelial cells. (**E**) Invasion of RMIT-O17 and Δ*lux*S-O17 to INT 407 epithelial cells showing the mutant exhibited significantly less invasion than the wild-type (*p* < 0.02). Invasion is expressed as the percentage of the inoculum that invaded the epithelial cells and showing a significant difference between the wild-type and mutant. (**F**) Invasive index of RMIT-O17 and Δ*lux*S-O17 to INT 407 epithelial cells. The invasion index is expressed as the percentage of the inoculum that invaded the epithelial cells divided by the cells adhered and showing significant difference between the wild-type and mutant. * *p* ≤ 0.05, *** *p* ≤ 0.001.

Biofilm formation by RMIT-O17 and Δ*luxS*-O17 was quantitated with CV assay (Figure 3A). The wild type produced a significantly higher amount of biofilm compared to the Δ*luxS* (*p* < 0.05) (Unpaired *t*-test). In each experiment, a negative control (no bacteria) was included to account for non-specific binding of the stain.

The motility of Δ*lux*S-O17 was compared to the wild-type. The results are presented in Figure 3B,C. Δ*luxS* showed reduced motility (approximately 70% of wild-type in BBA) (*p* < 0.02). The mean diameter of the spreading RMIT-O17 was 8.8 mm, and Δ*lux*S-O17 was 6.1 mm in semi-solid BBA.

The adhesion and invasion assays were performed for RMIT-O17 and Δ*luxS*-O17 on the INT407 epithelial cell line. The experiment was performed at a multiplicity of infection (MOI) of 100, as described previously. The proportion of *C. concisus* adherent or invading the cell line was calculated (Figure 3D,E). The invasion index was calculated using the formula described by Larson et al. [38] (Figure 3F). There was no statistically significant difference in the proportion of RMIT-O17 and Δ*lux*S-O17that were adherent to INT407 (Figure 3D). The mean % adhesion value of RMIT-O17 at MOI 100 was 0.13%, while the mean adhesion value of the Δ*lux*S-O17 was 0.087%. Interestingly, Δ*luxS*-O17 was significantly less invasive than the wild-type (*p* < 0.02). The invasion index of RMIT-O17 was 0.281, while for Δ*lux*S-O17 was zero. However, according to Larson et al. [38] a *C. concisus* strain that has an invasion index ≥ 1 was defined as an enteric-invasive *C. concisus* (EICC) strain. Yet, the wild-type RMIT-O17 was a non-EICC, because it’s invasion index was significantly low (*t*-test, *p* < 0.02) compared to Δ*lux*S-O17.

**Figure 4:**
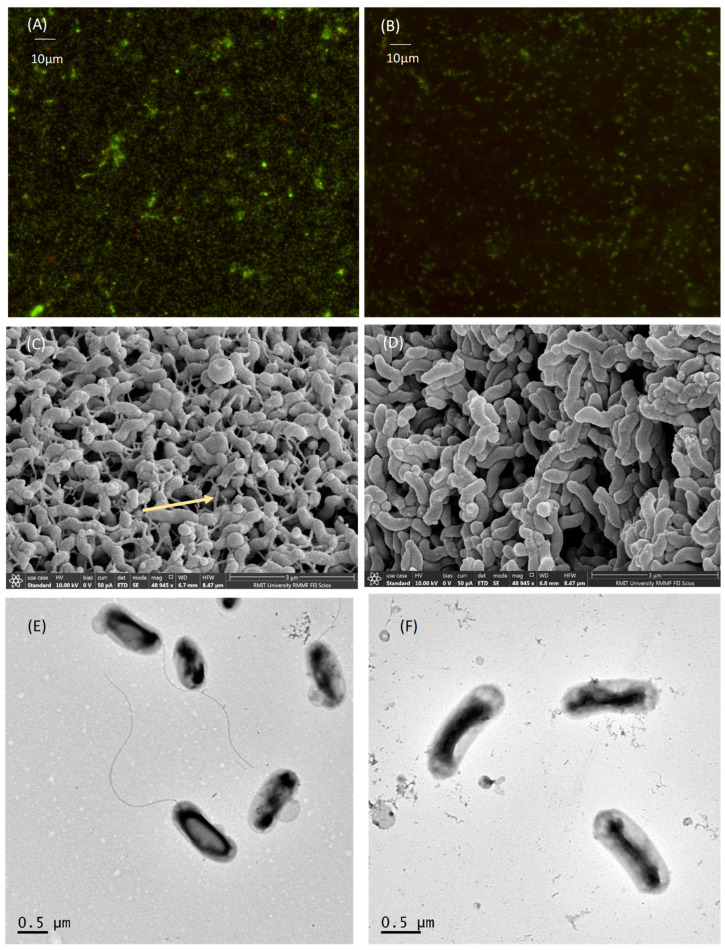
Biofilm of RMIT-O17 (**A**) and Δ*lux*S-O17 (**B**) by CLSM at 200x magnification showing less biofilm production by Δ*lux*S-O17 compared to the wild-type. Biofilm of RMIT-O17 (**C**) and Δ*lux*S-O17 (**D**) by SEM at 48,945× magnification showing less biofilm production by Δ*lux*S-O17 compared to RMIT-O17. Structures that looked like a flagellum (indicated by yellow arrow) were seen with RMIT-O17 (**C**) less abundance of that in the mutant was observed in the biofilms. TEM images showing RMIT-O17 (**E**) with the presence of a polar flagellum attached to the cell, and Δ*lux*S-O17 (**F**) lacking the flagellar structures.

For characterization of biofilm formation by microscopy, RMIT-O17 and Δ*lux*S-O17 were grown on coverslips in 6-well plates in Brucella broth for 4 days. Using CLSM, the RMIT-O17 biofilm on day 4 was seen to be a complex of live and a few dead cells present within small clumps, while no similar structures were found for the Δ*luxS* -O17biofilm (Figure 4A,B). All the cells of Δ*lux*S-O17 attached to the coverslip as single cells, and only live cells were seen in the biofilm.

Both strains were grown on 10 mm round coverslips in 12-well plates in Brucella broth for 4 days and observed with scanning and transmission electron microscopy. The biofilm of the wild-type RMIT-O17 on day 4 was in large clumps of cells present within aggregations attached to each other. Tubular criss-crossed network-like structures, probably flagellum in the biofilm, were clearly visible. No similar structures were found for the Δ*lux*S-O17 strain biofilm (Figure 4D). Cells of Δ*luxS*-O17 were flagella-free or defective structured. This finding is correlated with less motility and, hence, loss of invasiveness of the mutant compared with the wild-type.

In transmission electron microscopy, the wild-type RMIT-O17 was seen with a polar flagellum at one end of the body (Figure 4E), while the body structure of Δ*luxS*-O17 looked crooked and there was no flagellum seen at any end of the cell (Figure 4F). The absence of flagellum in TEM is also supported by the images found using SEM.

### 3.6. Expression of Flagellin Gene

**Figure 5:**
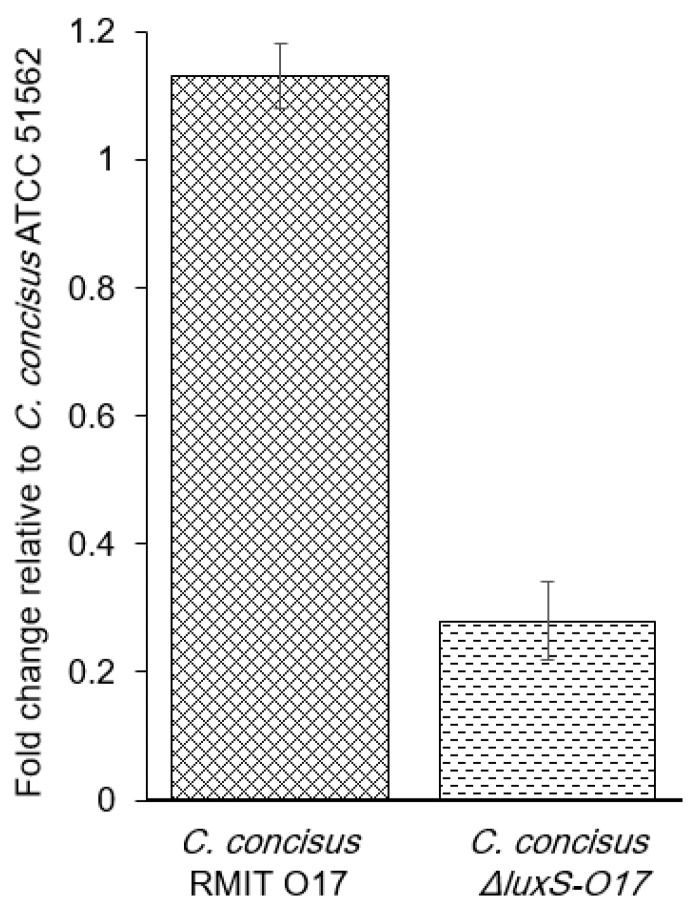
RT-qPCR of the *flaB* mRNA expression in *C. conci*sus RMIT-O17 and Δ*lux*S-O17 strain. Expression was normalised to the *atpA* and expressed as relative to the *C. concisus* ATCC 51562 reference strain. The expression of the *flaB* was significantly downregulated in the Δ*lux*S-O17 strain (dotted bar). Experiments were performed three times in triplicate, and error bars represent the standard error of the mean.

The *flaB* mRNA was detected by RT-qPCR in *C. concisus* ATCC 51562 as a control strain and in *C. concisus* RMIT-O17 and Δ*lux*S-O17 as the test strains (Figure 5). Mean Cq values of housekeeping gene *atpA* were 17.30, 17.26, and 18.33, respectively. Mean Cq values of *flaB* were 22.72, 22.49, and 25.58, respectively. The expression was normalised to the *atpA* and expressed as relative to the *C. concisus* ATCC 51562. The expression of the *flaB* was found to be significantly downregulated in the Δ*lux*S-O17 strain compared to RMIT-O17 (*t*-test, *p* < 0.0001).

## 4. Discussion

This study provides the first evidence of the role of *luxS* in biofilm formation by the bacterium *C. concisus*. Biofilm formation is often related to bacterial pathogenesis [12,39,40]; hence, it indicates that *luxS* is likely to influence *C. concisus* pathogenesis. Information on the pathogenic determinants of *C. concisus* is limited despite its pathogenic potential, as molecules possibly involved in biofilm formation have not previously been examined. In this study, *C. concisus* isolates from intestinal and oral sources were screened for biofilm formation. PCR of the genomic DNA of these isolates revealed the presence of the *luxS* in all.

Biofilm production was investigated using the quantitative crystal violet assay with minor modifications [23]. This assay is widely used for the detection of biofilm-forming ability of *Campylobacter* spp. and other oral pathogens [9,41,42]. Our study demonstrated that oral isolates produced significantly higher levels of biofilm than the intestinal isolates (*p* < 0.05). Ovesen et al. [11] reported similar results, with oral *C. concisus* isolates producing more biofilm than those isolated from gut mucosa or faeces. This may indicate that higher rates of biofilm formation could be an advantageous trait for colonization of the oral cavity, perhaps allowing escape from toxic oxygen and other adverse conditions. *C. jejuni* NCTC 11168 has been shown to develop biofilm more rapidly under environmental and food-chain-relevant aerobic conditions (20% O_2_) than under microaerophilic conditions (5% O_2_, 10% CO_2_) [41]. Interestingly, the first biofilm study of *C. concisus* by Gunther and Chen [9] was performed with an oral isolate, *C. concisus* ATCC 33237^T^, and it was found to produce more homogeneous biofilms compared to other *Campylobacter* spp. Lavrencic et al. [10] tested eight *C. concisus* strains for biofilm formation (all isolated from stool samples, including *C. concisus* ATCC 51561 and ATCC 51562) and reported that all eight strains were able to form biofilms. Our results also detected a moderate level of biofilm formation by *C. concisus* ATCC 51561 and ATCC 51562, in accordance with the previous study.

Direct microscopic observation revealed that *C. concisus* biofilms displayed clear developmental stages over the course of the study. This was used to conveniently partition biofilm development into three stages: (i) attachment, (ii) maturation, and (iii) dispersion. The developmental life cycle comes full circle when dispersed biofilm cells revert to the planktonic mode of growth. This mode of development in biofilms has been shown previously in *Pseudomonas aeruginosa* [43]. In our study, the mature *C. concisus* biofilm was found to be an aggregation of both live and dead cells, which is a characteristic of biofilms reported for other organisms. For example, a *P. aeruginosa* artificial biofilm was found to contain 13% dead cells after 8 h of incubation [44]. However, this scenario could vary depending on the type of biofilm and its participating members. *C. jejuni* has been reported to remain culturable for a longer time in a mixed biofilm with *P. aeruginosa* than in a monoculture biofilm [45]. The SEM image of *C. concisus* RMIT-O17 revealed the presence of an extracellular polymeric substance (EPS). This EPS helps the bacteria to adhere and, in other studies, it has been shown to consist of different chemical components, including exo-polysaccharides, proteins, eDNA (environmental DNA), and other polymers, which may provide protection from antibiotics and host defences [46]. The EPS has been identified in biofilms of other bacteria, such as *C. jejuni* [13], *P. aeruginosa* [46], and *Borrelia burgdorferi* [47]. However, no thorough investigation has been conducted to determine the composition of the EPS found in *C. concisus* biofilms.

LuxS is a signalling protein that has been linked to biofilm formation of many oral pathogens, such as *Streptococcus pneumoniae* [19], *S. mutans* [48], and *S. intermedius* [16]. The *luxS* or its homologue has been identified in *C. jejuni*, *C. coli*, *C. upsaliensis,* and *C. fetus*, but no *luxS* or homologue was identified in the *C. lari* genome [25]. Reeser et al. [23] found that both quorum-sensing and flagella are required for maximum biofilm formation by *C. jejuni*. Furthermore, Plummer et al. [24] analysed *luxS* sequence in *C. jejuni* 81116 and suggested that a mutation at amino acid position 92 (glycine to aspartic acid) in that gene was responsible for the loss of AI-2 activity (approximately 100-fold reduced catalytic activity).

In this study, *luxS* was identified with PCR in the genome of all *C. concisus* tested strains. However, our data showed that biofilm formation was significantly associated with the site of isolation, as oral cavity isolates comparatively produced higher levels of biofilms in vitro than intestinal isolates. Therefore, further investigation was performed to determine the role of *luxS* in *C. concisus* biofilm formation and other virulence-related characteristics.

A *luxS* knock-out mutant (Δ*lux*S-O17) was generated in *C. concisus* RMIT-O17, the highest biofilm producer among our tested isolates. Whole genome sequencing was performed on the knock-out mutant to confirm that the kanamycin cassette was correctly inserted in the targeted gene but not into other functional genes, which may lead to the disruption of other essential functions or metabolic activity. All other genes related to the flagellar structure and function were also checked in the annotated mutant’s genome sequence and compared with the wild-type’s genome to confirm the absence of any kind of manipulation in the mutant’s genome.

Biofilm formation by Δ*lux*S-O17 was found to be significantly reduced (*p* < 0.05), which supports *luxS’s* role in biofilm formation in *C. concisus*. The reduction of biofilm was also supported by the CLSM and scanning electron micrographs (SEM). Recently, a similar study in *C. jejuni* compared a wild-type and a *luxS* mutant and showed decreased motility, adhesion to polystyrene surfaces, and invasion of INT407 cells [49]. An association between AI-2 signalling and biofilm formation has been previously reported in *Streptococcus gordonii* [50], *S. mutans* [15,49], *Streptococcus anginosus* [51], *Klebsiella pneumoniae* [52], and in *H. pylori* [53]. Previously, Merritt et al. [49] reported a *luxS* mutant to be defective in AI-2 signalling; hence, quorum-sensing and biofilm produced by the *S. mutans luxS* mutant were different from the parental strain. It was also reported by Yoshida et al. [15] that biofilm formation of the *luxS* mutant in the same species was markedly decreased compared to the wild-type.

Studies on *C. jejuni* biofilms by Reeser et al. [23] found that both flagella and quorum-sensing are required for maximum biofilm formation, as both *C. jejuni flaAB* and *luxS* mutants were significantly impaired in their ability to form biofilms compared to the wild-type strain. In contrast, Lavrencic et al. [10] studied the phenotypic properties of eight *C. concisus* biofilm-forming strains isolated from gastrointestinal origins and did not find any correlation between motility and biofilm formation in *C. concisus*, but molecular investigation was not performed. The researchers concluded that motility is strain-specific, and it was suggested that those with higher motility have a greater chance to swim through the intestinal mucus layer, reach the epithelial surface, and cause diseases. In another study, Ovesen et al. [11] investigated the association between GI disease, motility, and biofilm-forming capacity in *C. concisus* and found no association, suggesting a negative correlation between motility and biofilm formation.

Our data from the genetic manipulation experiments of *lux*S showed that Δ*lux*S-O17 was comparatively less motile on the semi-solid agar medium than the wild-type RMIT-O17. Motility is directly related to flagellar function and structure; therefore, the possible absence of flagella-like projection in the mutant’s biofilm structure, as observed in micrographs obtained with SEM and TEM, may explain the decreased motility of Δ*lux*S-O17. As previously mentioned, we have confirmed from the genome sequencing data of Δ*lux*S-O17 that the kanamycin cassette was only inserted within the targeted gene/site. The expression level of *flaB* was assessed using RT PCR in both the wild-type and mutant strains, which indicated a significant reduction in the gene’s expression in the mutant. In *Vibrio alginolyticus,* LuxS was found to regulate flagella biogenesis [54], thus also regulating motility in *V*. *alginolyticus.* In *V. harveyi,* a motility assay and analysis of gene expression indicated the involvement of the quorum-sensing system, and autoinducer synthase mutants (Δ*luxO*) showed significantly lower swimming motility and expression of flagellar genes than the wild-type, which was restored by adding synthetic signalling molecules [26]. In *C. jejuni,* inactivation of LuxS also decreased motility on semi-solid media, suggesting a role for quorum-sensing in the regulation of motility [55]. The authors suggested that this system serves as a global regulatory mechanism for basic physiological function and virulence factors. Consistent with previous studies, ref. [56] reported that a *C. jejuni luxS* mutant had comparable growth rates to the parental strain and exhibited decreased motility halos in both MEM-alpha (Minimum Essential Medium Eagle Alpha modification) and MHB. Furthermore, Adler et al. [36] reported that different phenotypes of the *C. jejuni* Δ*luxS* depended on strain background, mutation strategy, and culture conditions. The researchers also complemented *luxS* with synthetic AI-2 or homocysteine, as well as the combination of both, and applied genetic complementation to partially restore the swarming ability of *C. jejuni* 81–176Δ*luxS*. Teren et al. showed that deletion of *luxS* in *C. jejuni* 81–176 results in a decrease in biofilm mass, and complementation of the gene in the mutant strains resulted in restored ability to produce AI-2, to form a complex biofilm at the level of the wild-type [55]. In our study, we constructed a *luxS* complementary plasmid to check whether the virulence properties can be reverted into mutant, however, after many attempts we were unable to successfully insert a complementary shuttle plasmid containing *luxS*. Probably, the size of the complementary plasmid was too large for the mutant strain to uptake.

Furthermore, unlike RMIT-O17, the Δ*lux*S-O17 mutant was found to be non-invasive in the INT407 intestinal cell line, although there was no significant difference in adhesion between the mutant and wild-type. *C. concisus* flagella plays a very important role in the invasiveness of the organism [57]. The defective invasion capabilities of the mutant strain can be related to the potential loss of flagellar structures in the mutant, which was observed by microscopy and gene expression assays. Mutagenesis of the *luxS* in *C. jejuni* has been found to completely disrupt the activated methyl cycle (AMC) with altered concentrations of AMC metabolites both upstream and downstream of LuxS [28]. Similar results were also found where a *C. jejuni* Δ*luxS* had comparable growth rate, resistance to oxidative stress, and ability to invade Caco-2 cell monolayers when compared to the parental strain [27]. However, the authors could not find any significant difference between the wild-type and mutant regarding invasion in Caco-2 monolayers. On the other hand, Quinones et al. [58] found significantly reduced colonization of the chicken lower gastrointestinal tract, reduced chemotaxis toward organic acids, and in vitro adherence to LMH chicken hepatoma cells after inactivation of *luxS* in *C. jejuni* strain 81–176. It was reported that AI-2 production in *C. jejuni* contributes to host colonization and interactions with epithelial cells. LuxS plays a role in *E. coli* O157:H7 in attaching and effacing lesions in pigs [59]. Inactivation of the quorum-sensing regulator in *V. vulnificus*, a food-borne pathogen, caused reduced virulence and colonization capacity in the infecting mice. Moreover, the mutant exhibited significantly reduced biofilm detachment to INT 407 host cells [60].

## 5. Conclusions and Future Directions

Our findings related to biofilm formation support the natural adaptation of this bacterium to the oral cavity environment. Hence, it can be concluded that *C. concisus* oral strains are higher biofilm producers than the intestinal strains, which could be a survival mechanism in their normal habitat. Furthermore, our data support the importance of LuxS and its role in biofilm formation and related virulence properties.

As a significant difference was observed between the biofilm levels of oral and intestinal *C. concisus*, a thorough investigation of the genes expressed during biofilm construction should be undertaken to better understand the relevant mechanism. A 3-dimensional view of the wild-type and the mutant biofilm using CLSM would provide insight into biofilm architecture. Other genes that could also influence biofilm formation, such as the flagellin gene, should be investigated. Future studies in animal models with Δ*lux*S-O17 and the parental strain are of importance to confirm the role of LuxS in pathogenesis.

## Figures and Tables

**Table 1:** Primers used in this study (5′-3′).

Primer Name	Size (bp)	Primer Pair	Primer Sequences	Template	Product size (bp)	T_A_ (°C)	Target gene	Source
FCCluxS RCCluxS	22 22	1	GAAACCATCTAAACGGCAACGG GTCCCATAGCATCAACGTCAAG	*C. concisus* gDNA	309	55	*luxS*	This study
luxScfc	30	2	GATCGGTACCATGAGCCTTCTTGCRGTRTC	RMIT-O17	1380	52	*luxS*	This study
luxScr1	30	2a	GCTAGAGCTCATAGAAGCGGCTCGTGCAGG					
luxScr2	31	2b	GCTAGAGCTCCAAGTCTCGCAGCCTAGAAAG					
Inv F O17	28	3	CGTAGGATCCGCCCATCGGTGAGATGTC	pBK *luxS*	3400	54	pBluescript containing *luxS*	This study
Inv R O17	33		GAGCGGATCCTCTACGCCGTTGCCGTTTAGATG					

Primers were named according to the gene’s name with a suffix letter ‘f’ or “r”. The letter “f” indicates forward primer and the letter “r” indicates reverse primer and R = Ag.

## Data Availability

The data that support the findings of this study are in this published article and available from the corresponding author upon reasonable request.
